# Analysis of Platen Superheater Tube Degradation in Thermal Power Plants via Destructive/Non-Destructive Characteristic Evaluation

**DOI:** 10.3390/ma15020581

**Published:** 2022-01-13

**Authors:** Se-Beom Oh, Jongbeom Kim, Soon-Woo Han, Kyung-Mo Kim, Dong-Seok Yun, Dong-Wook Kim

**Affiliations:** 1 Materials Safety Technology Development Division, Korea Atomic Energy Research Institute, Daejeon 34057, Korea; sbfull1269@gmail.com (S.-B.O.); swhan@kaeri.re.kr (S.-W.H.); kmkim@kaeri.re.kr (K.-M.K.); 2 Technical Expertise Research Center, Korea East-West Power Co., Ltd., Daejeon 35377, Korea; yds0917@ewp.co.kr (D.-S.Y.); ukvill@ewp.co.kr (D.-W.K.)

**Keywords:** thermal power plant, platen superheater, boiler tube, X20, ultrasonic non-destructive evaluation, tensile test, microstructural analysis

## Abstract

Coal-fired power plants operating under Korea’s standard supercritical pressure operate in a high-temperature environment, with steam temperatures reaching 540 °C. A standard coal-fired power plant has a 30-year design life, and lifespan diagnosis is performed on facilities that have operated for more than 100,000 h or 20 years. Visual inspection, thickness measurements, and hardness measurements in the field are used to assess the degree of material degradation at the time of diagnosis. In this study, aging degradation was assessed using an electromagnetic acoustic transducer to measure the change in transverse ultrasonic propagation speed, and the results were compared to microstructural analysis and tensile test results. Based on the experimental results, it was found that the boiler tube exposed to a high-temperature environment during long-term boiler operation was degraded and damaged, the ultrasonic wave velocity was reduced, and the microstructural grains were coarsened. It was also confirmed through tensile testing that the tensile and yield strengths increased with degradation. Our findings prove that the degree of change in mechanical properties as a function of the material’s degradation state is proportional to the change in ultrasonic wave velocity.

## 1. Introduction

Since the late 1980s, the design of coal-fired power plants has been standardized in the Korea as a strategy for building high-quality, large-scale power plants in a short period of time, laying the groundwork for self-reliance in comprehensive design and manufacturing technologies for power plants [1]. As a result, between 1993 and 2001, a total of 20 Korean standard supercritical pressure 500 MW standard coal-fired power plants have been built and are operational. With a steam pressure of 255 kg/cm^2^ entering the turbine and a steam temperature of 541 °C, supercritical pressure standard coal-fired power plants operate at high temperatures and pressures. The boiler pressure tube is composed of an economizer, a water-cooling wall, a superheater, and a reheater, and the materials used differ depending on the steam temperature and pressure in each section [2,3,4]. The superheater, in particular, is a facility that generates superheated steam by heating steam evaporated from the boiler’s water-cooling wall to raise the steam temperature, and it operates at high temperatures and pressures in the boiler tube. The reason for this is that the power plant’s thermal efficiency improves as the steam pressure and temperature increase [5]. As the superheated steam is used, the turbine’s thermal drop increases, its internal efficiency increases, the friction loss between the turbine and the steam supply pipe decreases, and the erosion by moisture is reduced, allowing it to operate at high temperatures and pressures [6]. Meanwhile, the design life of a supercritical standard coal-fired power plant is 30 years, and after 20 years of operation the aging process accelerates, requiring power plant life management [7].

The goal of coal-fired power plant life management is to increase revenue from power generation by improving performance and extending the lifespan of aging and degradation facilities. This is accomplished by regularly conducting lifespan diagnosis on facilities with equivalent operating hours (EOS) exceeding 100,000 h or that have been in operation for more than 20 years. By carefully diagnosing and replacing each component, the service life can be extended [8]. Visual diagnosis, thickness measurements, hardness measurements, and microstructure analysis in the field are currently being used to evaluate the degradation of boiler tubes in thermal power plants. The method of directly observing the microstructure by destroying high-alloy steel tubes is primarily used to confirm the degradation of tubes in thermal power plants. This method can be evaluated through comparison with the as-received materials. At a tempering temperature of 500~550 °C or higher, the hardness changes as the tube material being studied coarsens, and Cr-based carbide forms as the grain boundary grows. As the carbide coarsens, microstructural analysis is required to confirm that the hardness changes as the obstacles preventing dislocation movement decrease [9,10,11,12,13,14].

As destructive tube analysis is costly and has time constraints, non-destructive methods are being investigated. Internal deformation or voids caused by material degradation cause diffuse reflection and diffraction of ultrasonic signals, resulting in incomplete signal transmission during transmission and reception. Because this principle causes changes in ultrasonic wave velocity, signal amplitude attenuation, non-linear parameter changes, and others, it can be used to determine the degree of material degradation [15,16,17,18]. Nam et al. [19] used ultrasonic signal attenuation to assess the properties of fatigue cracks in welds. Piloix et al. [20] used the ultrasonic damping coefficient to assess the degradation of stainless-steel welds. Aghaie-Khafri et al. [21] used ultrasonic attenuation evaluations to assess the grain size and yield strength. Zeng et al. [22] determined how much ultrasonic attenuation occurs at grain boundaries after annealing. Finally, Ohtani et al. [23] investigated the ultrasonic damping caused by low-carbon steel fatigue crack generation. However, there are only a few empirical studies that use ultrasonic signal characteristics to directly measure the degree of long-term degradation of boiler tubes in thermal power plants.

In this study, textural analysis was carried out by sampling platen superheater (PSH) tubes that had been in use for approximately 20 years after the completion of the thermal power plant and were considered to be in poor condition. Ultrasonic inspection (velocity measurements) was used to compare and analyze the degree of degradation of the material. Three different locations were used to collect samples of actual boiler tubes that had long-term use, including the first unused specimens of the PSH tube of the thermal power plant. Microstructural analysis was performed on each sample to confirm the change in microstructural grains and a tensile test was performed after measuring the ultrasonic propagation velocity. The rates of change as a function of degradation, tensile strength, and yield strength were measured and compared.

## 2. Supercritical Tubes in Standard Coal-Fired Power Plant Superheaters (PSH)

**Figure 1:**
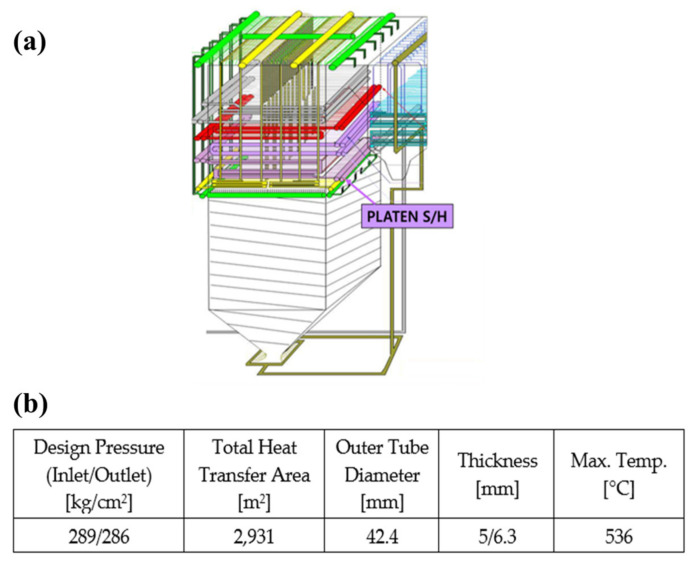
(**a**) Overview of a Korean standard coal-fired power plant PSH, (**b**) operating conditions.

Figure 1 shows the detailed specifications of the standard coal-fired power plant PSH tube used in this study. The initial design specifications are as follows: design pressure (inlet/outlet): 289/286 kg/cm^2^; total heat transfer area: 2931 m^2^; outer tube diameter: 42.4 mm; thickness: 5/6.3 mm; and maximum temperature: 536 °C. The X20CrMoV12.1 PSH (500 MW USC super heater boiler, Korea) was developed for use in high-temperature and high-pressure power plants as a type of high-alloy steel with a high Cr content, which strengthens its corrosion resistance. The maximum operating temperature of a thermal power plant in the Korea is 545 °C, with 450 tubes. The tube used in the experiment ran for approximately 167,474 h, and samples were taken from the boiler, which was started 89 times.

Because PSH tubes in standard coal-fired power plants operate at high temperatures and pressures, X20CrMoV12.1 was used, which has excellent high-temperature oxidation resistance and creep strength characteristics. DIN X20CrMo12.1 steel, which is used in standard coal-fired power plant boiler tubes, contains 12% Cr and additional W or Mo to improve its high-temperature strength, allowing it to be used at temperatures as high as 580 °C. Additionally, it has good resistance to repeated thermal stress, as its thermal expansion coefficient is low and the change in the heat-transfer coefficient as a function of temperature is small. Due to the high amount of Cr, it has excellent corrosion resistance, and the scale formation temperature can reach 700 °C. However, its ductility tends to decrease as the creep rupture time increases according to its properties [24,25]. The chemical composition of the generally used boiler tube is shown in Table 1.

**Figure 2:**
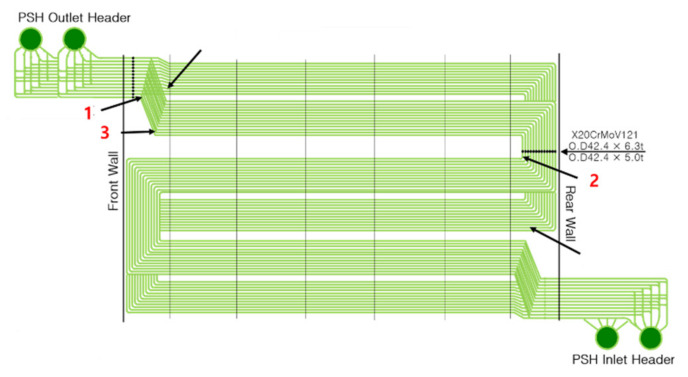
PSH schematic diagram and tube sample location.

**Figure 3:**
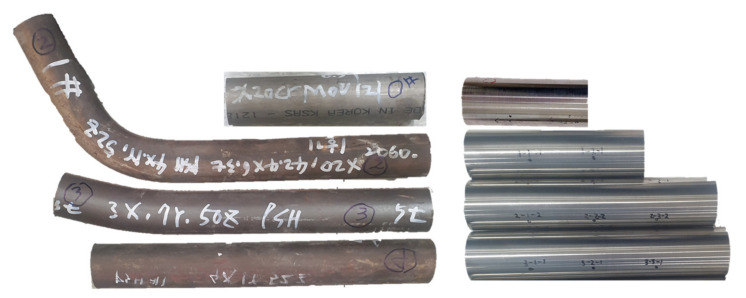
Boiler tube original samples (**left**) and after processing for ultrasonic measurement (**right**).

For initial reference, a sample of the same material that was not directly used in the power plant was set as Specimen No. 0, and specimens were collected from three PSH locations, as shown in Figure 2. Specimens 0, 1, and 3 were 6.3 mm thick, while Specimen 2 was 5 mm thick, and the operating temperatures at each location are indicated in Table 2. The straight pipe area was cut from the removed sample tube to increase the reliability of the ultrasonic measurement results. To obtain a uniform roughness across all specimens, surface treatment was used to remove corrosion products caused by aging, as shown on the right of Figure 3.

## 3. Experimental Method and Results

### 3.1. Tube Analysis along the Circumferential Direction

**Figure 4:**
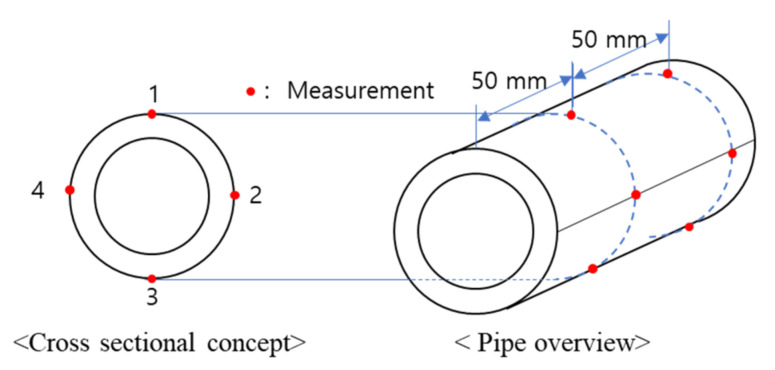
Location of the ultrasonic velocity and microstructural analysis measurements.

To analyze the difference according to the tube installation location direction, four measurement points were set at 90° intervals in the circumferential direction, and the ultrasonic velocity was measured at 50 mm intervals in the axial direction, as shown in Figure 4. A cross-section in the same circumferential direction as the ultrasonic measurement position was secured for surface grain analysis at the corresponding position, and the surface was analyzed using a microscope (VHX-5000, KEYENCE, Osaka, Japan) Following the ultrasonic velocity measurements at the reference point, a tensile specimen was prepared at the same location, and the tensile and yield strengths were assessed.

### 3.2. Ultrasonic Wave Velocity Measurement

**Figure 5:**
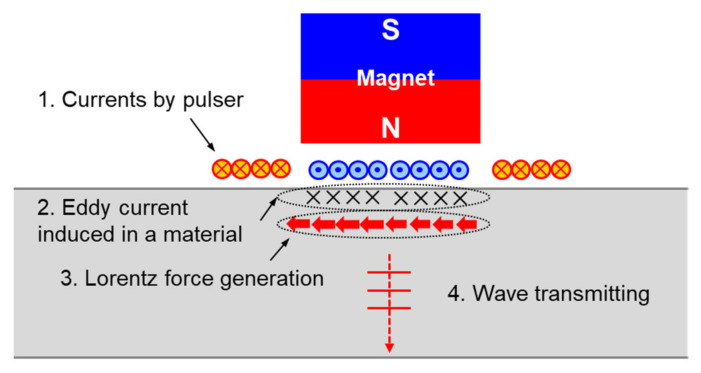
Ultrasonic wave generation mechanism of the EMAT used in the experiments.

**Figure 6:**
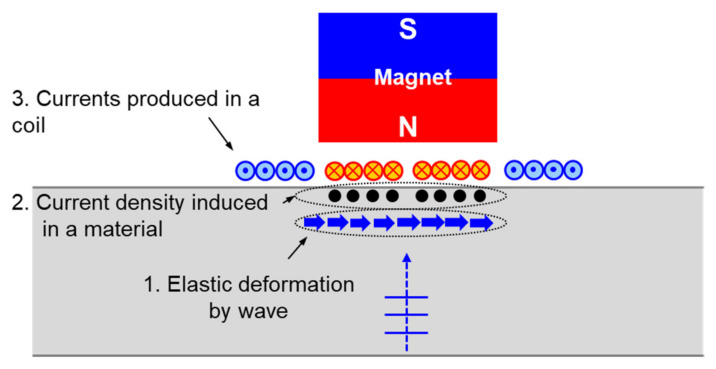
Ultrasonic wave receiving mechanism of the EMAT used in the experiments.

An electromagnetic acoustic transducer (EMAT) (FOE-SV1M, Sensysco, Sejong, Korea), which was photo-etched on a flexible printed circuit board (FPCB)) (FOE, Sensysco, Sejong, Korea), and manufactured as a coil, was used to reduce measurement deviation due to pipe curvature and increase the reliability of the measurements. EMAT, as is well known, uses a combination of electric current and magnetic field to generate and measure an elastic wave in a conductor. The following are the EMAT elastic wave generation and measurement methods used in this study. As an eddy current is formed on the surface of the measurement target when a coil is installed on the upper part of the pipe and alternating current flows through it, a Lorentz force is generated due to the permanent magnet generating a vertical magnetic field in the conductor in a direction perpendicular to both the eddy current and the magnetic field, resulting in the creation and propagation of an elastic wave. The EMAT ultrasonic wave generation and receiving mechanisms are shown in Figure 5 and Figure 6, respectively.

Next is a description of how the EMAT was used to measure elastic waves in this study. Elastic deformation occurs in the portion of the conductor where the elastic wave generated in the conductor is reflected from the interface and returned to the vicinity of the coil. Then, a current density is generated in a direction that is orthogonal to both the elastic deformation and the magnetic field. As a result, the coil generates an electromotive force that can be used to measure the elastic wave.

**Figure 7:**
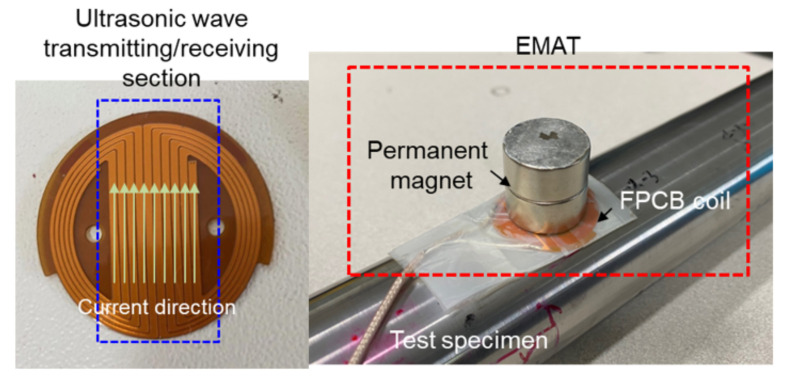
Coil and EMAT configuration used in the experiments.

Figure 7 shows the coil and EMAT configuration used in the experiments. On the FPCB, the coil was fabricated by photo-etching a circuit designed so that all currents in the center could flow in the longitudinal direction of the specimen. To generate a vertical magnetic field, a circular neodymium magnet (D20H10, Jeongwoo, Seoul, Korea), with a diameter of 20 mm and a height of 20 mm was installed on the coil.

**Figure 8:**
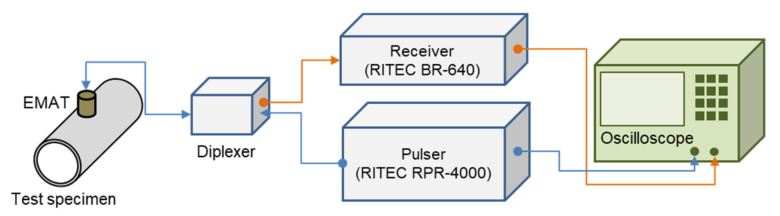
Schematic of the experimental setup.

Figure 8 shows the experimental setup of the ultrasonic velocity measurements. The reference signal was sent to the EMAT by a RITEC RPR-4000 high-current pulser (RPR-4000, RITEC, Warwick, RI, USA), and the signal received from the EMAT was amplified by a RITEC BR-640 receiver (BR-640A, RITEC, Warwick, RI, USA). A diplexer (RDX-6, RITEC, Warwick, RI, USA), was installed between the pulser/receiver and the EMAT to classify the transmitted and received signals for the pulse-echo method of signal transmission and reception. An oscilloscope (HDO-4024, LeCroy, Chestnut Ridge, NY, USA) was used to record the reference and received signals of the pulser and receiver, respectively, which were then analyzed on a computer. The reference signal is a 1-cycle sine-wave signal with a central frequency of MHz and a gain of 64 dB on the receiver.

**Figure 9:**
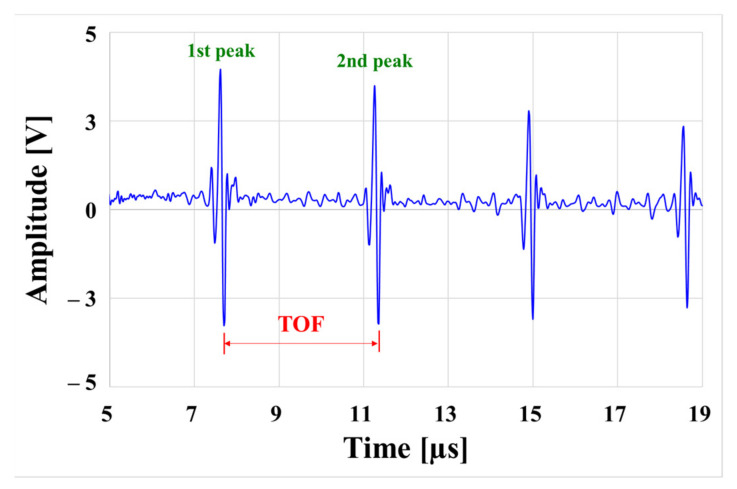
Ultrasonic shear wave measurement signal.

The pulse reflection method was used to obtain the ultrasonic signal received through the experimental device, as shown in Figure 9. The ultrasound propagation speed can be calculated using Equation (1) by calculating the difference in propagation time between the first and second peaks received in Figure 9.

(1) $$C\;\left[\frac{m}{s}\right]=\frac{2L\;\left[\mathrm{mm}\right]}{S\;\left[s\right]}$$

where *C* is the ultrasonic propagation speed, *L* is the specimen thickness (mm), and *S* is the time of flight (s).

**Figure 10:**
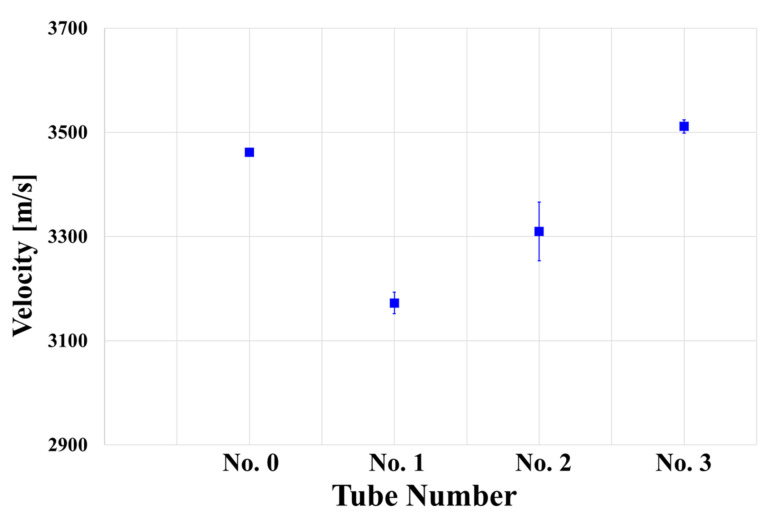
Ultrasonic velocity of the four tube specimens.

The ultrasonic propagation speed was measured and compared in both the circumferential and longitudinal directions of the specimen at the measurement points shown in Figure 4; a velocity difference of 10~20 m/s was found in each direction. The data at all measurement points were averaged and compared, as the deviation of the ultrasonic propagation speed compared to the material speed was insignificant (0.8%). The ultrasonic propagation speed (transverse wave) in each specimen, including Specimen No. 0, was measured and averaged, as shown in Figure 10. It was confirmed that the ultrasonic velocity of the age-related degradation specimens was generally slower than that of the initial specimen, especially in the case of Specimens 1 and 2. 

A difference in density caused by the movement and bonding of dislocations in the material, or a change in grains as a result of this in general, can change the ultrasonic velocity. The change in dislocation, in particular, causes deformation of the matrix lattice, which in turn alters the elastic or mechanical properties of the material [26]. The ultrasonic velocity is known to be physically dependent on the modulus of elasticity or closely related to the material density. Because the material density does not change significantly as a result of heat treatment, the change in ultrasonic velocity due to heat treatment is attributed to changes in the elastic properties in this study [27]. The lattice deformation is restored when the dislocation density decreases due to degradation. As a result, the elastic properties of the material change, and it can be inferred that the velocity decreases due to degradation damage [28,29,30,31]. Assuming that all degradation conditions are the same, we see that the ultrasonic velocity varies depending on the sampling location of the specimen. This means that the thermal effect may differ depending on the location of the tube inside the actual heater, even if the thermal conditions in the power plant system are the same.

### 3.3. Microstructural Analysis

**Figure 11:**
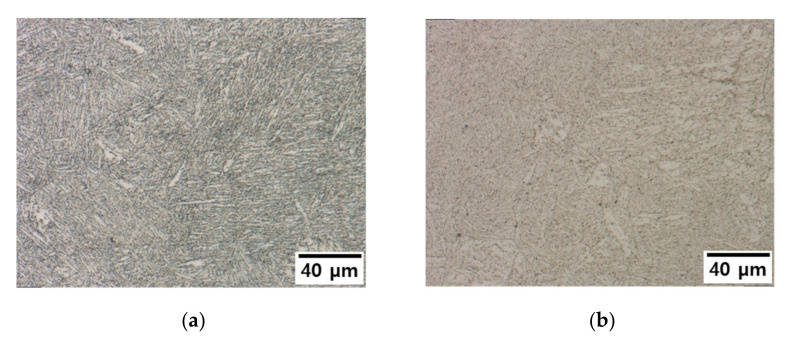

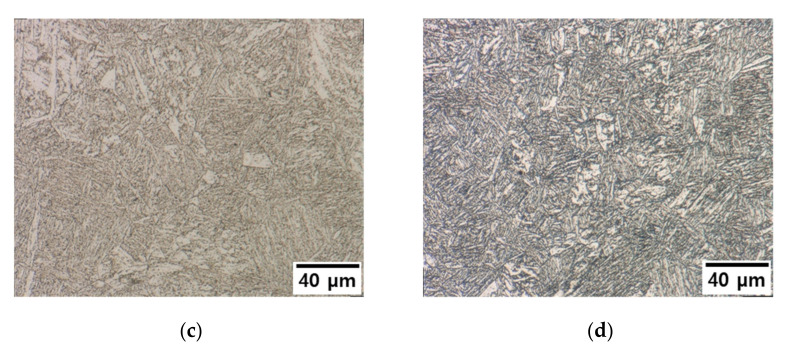
Microstructure according to tube type for specimens (**a**) No. 0, (**b**) No. 1, (**c**) No. 2, and (**d**) No. 3.

The surface was analyzed by cutting in the vertical direction of the tube circumference to observe the change in hardness as the carbide coarsened, reducing the obstacles blocking the dislocation movement. The surface was observed at 500-times magnification after etching for 1 min with a 5% Nital solution. A needle-shaped ferrite structure was confirmed to be dominant in the untreated initial specimen (No. 0) (Figure 11a). Some grains were enlarged in Specimens 1, 2, and 3 collected from the power plant, indicating that a cubic bainite structure had grown in comparison to the initial specimen (Figure 11b–d). When a tube is heated to a certain temperature, the tube undergoes microstructural changes. In this study, we observed that continuous thermal damage exceeding 500 °C causes fine grain bonding, resulting in structural changes. When the structure becomes coarse, the strength increases differently than when the structure degradation in general [32,33]. The etching time was different in Specimen 3 in particular. It was impossible to confirm grains and grain boundaries when etching was done at the same time as the other specimens; however, it became possible when more etching time was given. Due to material degradation, the ultrasonic propagation speed is assumed to be higher than that of the initial specimen (No. 0), while the surface or internal structural properties differ from those of other specimens. Generally, due to an increase in the precipitate size, carbon steel degradation is accompanied by an increase in hardness and a decrease in strength. Although the high alloy used in this study has better corrosion resistance than general carbon steel, we observed that the microstructure was altered by continuous high-temperature/high-pressure thermal curing for more than 160,000 h.

### 3.4. Tensile Test

**Figure 12:**
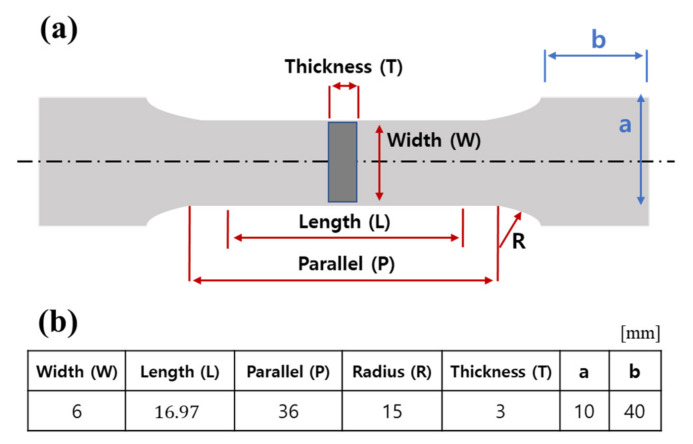
Specifications of the tensile test specimen, (**a**) overview, (**b**) specimen size.

**Figure 13:**
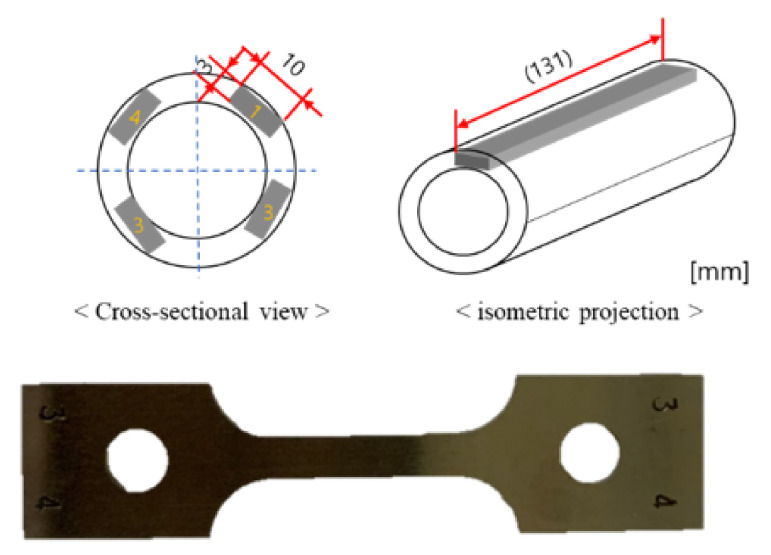
Extraction location and manufactured tensile test specimen.

A comparative analysis of tensile strength, yield strength, and elongation can be used to confirm that grain coarsening due to degradation can lead to changes in the mechanical properties of materials. The specifications of the tensile test specimen were determined using ISO 6892 standards, as shown in Figure 12. The tube was divided into 4 equal parts, similar to the ultrasonic measurement direction, and the specimen was cut as shown in Figure 13.

**Figure 14:**
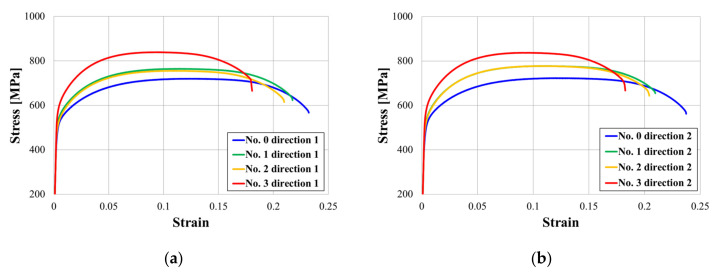

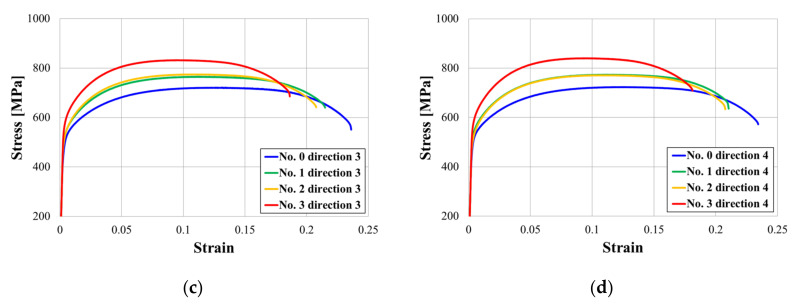
Tensile test results according to the circumferential direction in the four locations shown in Figure 4: (**a**) 1, (**b**) 2, (**c**) 3, and (**d**) 4.

**Figure 15:**
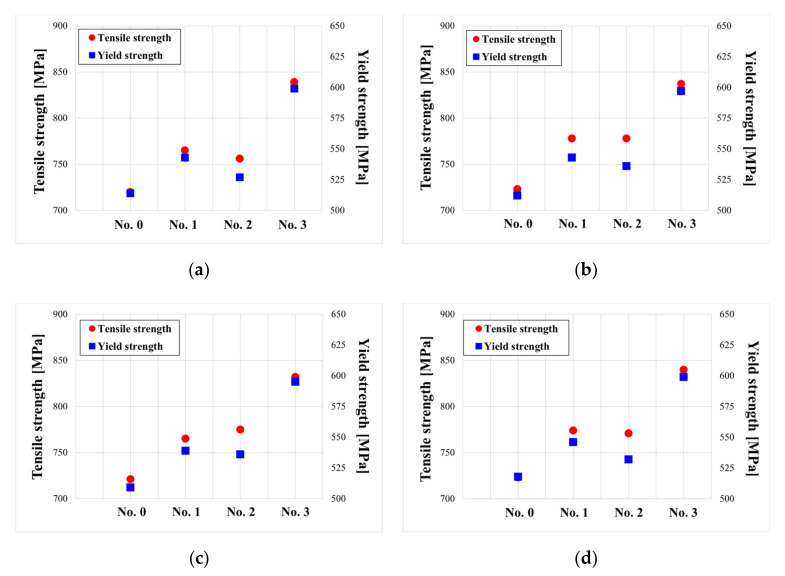
Tensile and yield strengths for the four specimens in the four directions shown in Figure 4: (**a**) 1, (**b**) 2, (**c**) 3, and (**d**) 4.

At room temperature, a tensile test was performed with a maximum load of 5 kN and a displacement rate of 0.1 mm/s (MTS Insight 50, Eden Prairie, MN, USA). The tensile test results for each specimen are shown in Figure 14. The specimen is divided into four directions at 90° intervals based on the circumferential direction of the pipe (Figure 14a–d). In each figure ((a) to (d)), the lines of the same color represent the same specimen but a different direction, and it was verified that the change tendency of the stress strain curve was similar regardless of direction. The fact that the strength change was unaffected by the direction of the test piece indicates that the heat effect in the tube installed in the power plant is unaffected by position in the circumferential direction. Furthermore, the ultrasonic velocity measurement results also showed little variation according to direction. As a result, the low deviation in the circumferential direction of the tube was confirmed. In addition, the phenomenon that stress acts a lot as strain decreases in the change in the graph means that the hardness increased as the ductility of the material decreased. Figure 15 shows the changes in the stress–strain curve according to the measurement direction of the tensile test results of the initial and damaged samples. The tensile and yield strengths in the tube at the remaining positions increased, while the elongation decreased, as compared to the initial specimen (No. 0). The strength of the sample according to the material increased similarly in Sample No. 1 and 2, and the strength of No. 3 increased significantly in the initial specimen (Figure 14). This trend of increasing tensile and field strength does not vary significantly depending on the direction of the specimen (Figure 15). On average in all directions, No. 1 and 2 showed an increase of about 50 MPa and an increase of 100 MPa or more in No. 3 compared to the initial specimen. These results show a trend similar to those observed at ultrasonic speeds, as shown in Figure 10. This is because the increase in tensile and yield strength is similar to the gradual increase in ultrasonic speed from Specimens No. 1 to 3. However, although the ultrasonic velocity of Specimen No. 3 was nearly identical to that of the initial specimen, its tensile test result was significantly different from that of the initial specimen. This can be related to the microstructural analysis results. While grain coarsening was mostly visible in Specimens No. 1 and 2, after grain coarsening, the shape of Specimen No. 3 reverted to a dendrite structure, and the color of its surface changed as well. Due to a precipitate size increase, carbon steel degradation is accompanied by an increase in hardness and a decrease in strength. Although the high alloy used in this study has better corrosion resistance than general carbon steel, it is believed that its microstructure was altered by continuous high-temperature/high-pressure thermal curing for more than 160,000 h, and the mechanical properties are believed to be affected by changes in the material properties under certain conditions.

## 4. Conclusions

This study is significant not only because it directly evaluates boiler tubes with a long-term degradation time (160,000 h or more), but also because the specimens used in the study were directly sampled on-site in the thermal power plant PSH, where they are considered to be at their most vulnerable due to aging degradation. The purpose of this study was to suggest a test method that could be used to confirm the reliability of existing methods or to devise a new test method using ultrasonic non-destructive testing. The ultrasonic velocity is constant owing to the properties of the material; however, when degradation or defects occur, the elastic properties of the material are minutely altered due to microstructural and internal changes, resulting in a change in the ultrasonic velocity. If the technique for measuring this change is sufficiently verified, the degree of degradation can be determined without having to remove the tube from the field. To support the analysis results, the ultrasonic velocity was measured and compared based on the tube position and initial undamaged specimen (Specimen No. 0). The microstructural images were taken, and the mechanical properties were evaluated through tensile testing. As a result of the ultrasonic velocity measurements, the grain size was bigger as the material degraded, and as the material hardened, the ultrasonic velocity decreased and changed according to the characteristic variation of degradation. This means that changes in the mechanical properties of the material caused by variations in ultrasonic velocity can be measured. However, if the structural form of some materials is altered outside of their degradation range, and the elastic properties of the material revert to those of the initial specimen, it is difficult to perform a precise degradation analysis because the ultrasonic velocity is measured similarly, requiring additional tests.

## Figures and Tables

**Table 1:** Chemical composition (wt.%) of boiler tube.

	C	Si	Mn	P	S	Ni	Cr	Mo	V
X20 CrMoV12.1	0.17~0.23	Max 0.50	0.40~0.70	Max 0.030	Max 0.030	0.30~0.80	10.00~12.5	0.80~1.25	0.25~0.35
P91	0.08~0.12	0.2~0.5	0.3~0.6	Max 0.02	Max 0.01	Max 0.4	8.0~9.5	0.85~1.05	0.18~0.25

**Table 2:** Operating temperature according to pipe location.

Specimen Number		1	2	3
Temperature [°C]	Max.	545.14	544.38	528.31
	Avg.	489.60	492.83	481.77
